# Periostitis

**Published:** 1831

**Authors:** 


### LXVII.

### Periostitis.

### [Meath Hospital.]

William Darcy, ætat. 32, a labourer, admitted April 5th, 1831. Complains of very severe pain in the right side of the head, extending down the side of the face and neck as far as two abscesses; one situated at the upper part of the sternum, not yet open, and of a dull red colour; the other at the internal third of the left clavicle, open, and in which a probe can be passed more than an inch and a half, discovering the bone to be carious. From the left side of this abscess a hard tender swelling extends for more than two inches along the clavicle, and a very short way up the neck.

All the right side of the head, face, and neck, is very tender, and in some places red and very slightly swollen, with a sensation of numbness. The pain in the head is constant, worse at night, and aggravated by cold or wet weather, or exposure to wind. He has no other pain. His history is as follows:—He had several attacks of the venereal disease, for which he was salivated two or three times. The last attack was six years ago. Since that time his health continued pretty good, without pain or eruption, till a year since, when he first experienced a severe pain about the middle of the sternum, with some degree of swelling, worse at night, and increased by pressure; and, a week or two after its first appearance, accompanied by a swelling every night in the throat and face, which went off in the morning. After several months he was relieved from this affection in this hospital.

At the time of the snow, about nine weeks ago, he caught cold, and pain was experienced at the left clavicle, and side of the neck; the pain leaving the left side, attacked the parts at present engaged with great severity; since which time he has been deprived of rest at night, and lost his strength and health. The abscess on the upper part of sternum appeared a fortnight since, preceded by pain in the spot. Appetite good; tongue loaded, red at tip; bowels confined; pulse slow.

Hir. xii. capitis parti sinistræ; et habeat decoct. sarsap. ℔i.; Acid nit. ʒi.

6th.—Little relief of the pain. Two needles were inserted about an inch deep into the temporal muscle, with great mitigation of the pain, though not its entire removal. Rep. decoct.

7th. He slept yesterday two hours after the insertion of the needles, and his head-ache for the rest of the day was not so severe; to-day, however, it is nearly as bad as ever. The abscess in the sternum was opened, and the opening in the one on the left clavicle enlarged. Rep. decoct. et applicetur vesicat. capiti.

The leeches and blisters were repeated, with the daily use of the decoct. sarsap. and occasional purgatives. By these means the pains became gradually less, and at present he has no pain any where, but only some slight degree of swelling and a sensation of numbness in the right side of the face (April 23d.) The abscesses, though not yet healed, are greatly improved in appearance, the centre granulating, and the parts are less red and swollen, and no longer tender.

Remarks.—The result of this case is very satisfactory, as, from the obstinacy and severity of the pain, little good was at first expected to be produced. At present the man is at least in a state of comparative ease from a very distressing affection. Some of these cases of periostitis in bad constitutions, and where the system has been shattered by repeated courses of mercury, assume a very severe form; the periosteum not only being painful and tender to the touch, but a semi-cartilaginous deposition taking place between the bone and periosteum, ending in suppuration and disease of the bone, as in this case. An abscess, however, frequently forms, without being preceded by the semi-cartilaginous deposition, from inflammation of the periosteum; such was the case in Graydon, the man with jaundice, under Dr. Graves. The semi-cartilaginous deposition appears, also, when occurring in good constitutions, to have a disposition not to run into suppuration, coming and going for a number of years with little change in size or situation. The pain is always most severe when this disposition exists, and is often of an intermitting character, being worse at night. So great was the agony in one woman with a swelling from this cause on the lower part of the tibia, that greater part of the night was passed in pacing up and down the ward. In her it was of many years' standing, and had been often treated. When abscesses form in broken constitutions, such as this man's, they are of a dull purplish red, sluggish, and from the diseased bone, very difficult of cure. The best way appears to be, to open them freely, dress from the bottom with red precipitate and lint, and on the appearance of granulations to touch with the sol. argent. nit. and apply a strap. The first favourable impression on the pain was by acupuncturation.—*Med. Gazette.*

---

LXVIII.

